# K Deprivation Modulates the Primary Metabolites and Increases Putrescine Concentration in *Brassica napus*

**DOI:** 10.3389/fpls.2021.681895

**Published:** 2021-08-13

**Authors:** Elise Réthoré, Lun Jing, Nusrat Ali, Jean-Claude Yvin, Sylvain Pluchon, Seyed Abdollah Hosseini

**Affiliations:** ^1^Laboratoire de Nutrition Végétale, Agro Innovation International—TIMAC AGRO, Saint-Malo, France; ^2^Plateformes Analytiques de Recherche, Agro Innovation International—TIMAC AGRO, Saint-Malo, France

**Keywords:** primary metabolites, amino acids, nutritional homeostasis, mineral nutrition transporters, electric charge balance

## Abstract

Potassium (K) plays a crucial role in plant growth and development and is involved in different physiological and biochemical functions in plants. *Brassica napus* needs higher amount of nutrients like nitrogen (N), K, phosphorus (P), sulfur (S), and boron (B) than cereal crops. Previous studies in *B. napus* are mainly focused on the role of N and S or combined deficiencies. Hence, little is known about the response of *B. napus* to K deficiency. Here, a physiological, biochemical, and molecular analysis led us to investigate the response of hydroponically grown *B. napus* plants to K deficiency. The results showed that *B. napus* was highly sensitive to the lack of K. The lower uptake and translocation of K induced *BnaHAK5* expression and significantly declined the growth of *B. napus* after 14 days of K starvation. The lower availability of K was associated with a decrease in the concentration of both S and N and modulated the genes involved in their uptake and transport. In addition, the lack of K induced an increase in Ca^2+^ and Mg^2+^ concentration which led partially to the accumulation of positive charge. Moreover, a decrease in the level of arginine as a positively charged amino acid was observed which was correlated with a substantial increase in the polyamine, putrescine (Put). Furthermore, K deficiency induced the expression of *BnaNCED3* as a key gene in abscisic acid (ABA) biosynthetic pathway which was associated with an increase in the levels of ABA. Our findings provided a better understanding of the response of *B. napus* to K starvation and will be useful for considering the importance of K nutrition in this crop.

## Introduction

Potassium is one of the most important macroelements for plant growth and development, and can represent up to 10% of plant dry weight (Wang and Wu, 2013). It plays a major role not only in the regulation of cell turgor pressure, pH, and electrical balance, in photosynthetic activity, and protein synthesis but also in the activation of numerous enzymes (Marschner, 2012). Although potassium (K) reserve accounts for 2.6% of the earth’s crust, its availability to plants in the soil solution represents a small proportion of total K in the soil (0.1–0.2%) (Sardans and Peñuelas, 2015). Moreover, in many arable lands, the current application of K is insufficient to cover its removal from crops, since K fertilization is usually underestimated compared to the application of nitrogen (N) and phosphorus (P) and only 35% of the used K is replenished in the field (Römheld and Kirkby, 2010; Zörb et al., 2014). In the context of climate change, the improvement of K nutrition is crucial because this element participates in the alleviation of abiotic stresses, such as drought or salt stress, particularly by regulating stomatal opening and maintaining osmotic balance (Hasanuzzaman et al., 2018). Thus, it is essential to better understand the effects of K limitation on plant development and metabolism.

*Brassica napus* is an oilseed crop and an important resource of edible oil which is necessary for humans (Chakraborty et al., 2016). For proper growth and good yield, it requires high levels of nutrients (Grant and Bailey, 1993), and deficiency in one of the nutrients imposes a yield penalty (Coleto et al., 2017). The role of N (Sorin et al., 2015), sulfur (S) (Zhang et al., 2020), or boron (B) (Pommerrenig et al., 2018) has widely been investigated in *B. napus*. Even though the response of many crops to K deficiency has been extensively studied over the past decades, little is known about the response of *B. napus* to K starvation. When encountering K deficiency, cytosolic K concentration is declined which influences on the activity of cytosolic enzymes. When K is deficient, growth is retarded, and the net transport of K from mature leaves and stems is enhanced. Under severe K deficiency, these organs become chlorotic (Marschner, 2012). Moreover, under K deficiency, a decrease in primary root growth and lateral root initiation is observed together with an increase in the length and density of root hair (Høgh-Jensen and Pedersen, 2003).

Over the last decades, multi-scale studies have elucidated the signaling pathways activated under K limitation as well as the subsequent metabolic adaptations occurring in plants. One of the first steps in the sensing of K deficiency is performed by Ca^2+^ channels in the root epidermis, which are activated by the hyperpolarization of the membrane (Wang et al., 2018). The consequent Ca^2+^ influx activates the downstream signaling pathway *via* calmodulins, CaM-like proteins, calcium-dependent protein kinases, and calcineurin B-like proteins to regulate K transporters, such as *AKT1* (Cheong et al., 2007; Wang et al., 2018). Ethylene was also shown to be involved in the signaling of K deficiency, acting upstream of reactive oxygen species (ROS) to increase the expression of the high-affinity transporter, *HAK5* and to regulate primary root growth and root hair elongation (Jung et al., 2009; Schachtman, 2015). Besides the involvement of ethylene, abscisic acid (ABA) was also shown to be involved in this stress response (Peuke et al., 2002; Nam et al., 2012; Wang et al., 2012).

Moreover, the K deficiency disturbs the uptake and assimilation of other elements in the plant. Indeed, under low K condition, an increase in the concentration of other cations, such as Na^+^, Mg^2+^, and Ca^2+^ occurs which is required to maintain the pH and the osmotic balance in the cell (Wakeel et al., 2011; Hafsi et al., 2014). In addition, K and N metabolism were shown to interact with each other, and this cross-talk was shown by the antagonism effect between ammonium (NH_4_^+^) and K and the co-operative uptake, transport, and utilization between nitrate (NO_3_^–^) and K (Wang and Wu, 2013; Coskun et al., 2017).

Plants also respond to low K by adapting to their metabolism. Based on a multi-level analysis of the primary metabolism in *Arabidopsis* under low K supply, Armengaud et al. (2009) concluded that the primary cause of metabolic disorders in low-K plants could maintain the metabolic flow toward amino acids and protein synthesis, while decreasing negative metabolic charges and increasing N/carbon (C) ratio in the amino acids (Armengaud et al., 2009). Moreover, the concentration of sugars was significantly increased and was correlated with the inhibition of pyruvate kinase activity by low K availability. Among the polyamines, putrescine (Put) was also specially shown to be accumulated in plants under K deficiency and is considered as a potential marker in response to K starvation. Accumulation of Put was proposed to be used as a strategy in order to control cation balance in the cell (particularly, Ca^2+^/K^+^ balance) and to protect energy metabolism (Cui et al., 2020b). Overall, the metabolic changes in plant exposed to K deficiency were conserved in diverse crops, like barley (Zeng et al., 2018), tomato (Sung et al., 2015), or cotton (Hu et al., 2017).

Different studies have focused on the effect of K deficiency on specific organs or physiological parameters in rapeseed plant (Lu et al., 2016, 2019; Tong et al., 2020). In this study, an effort was undertaken to investigate the overall response of *B. napus* to the lack of K nutrition. Therefore, the hydroponically grown plants were exposed to long- and short-term K starvation and a global overview of its response to K deprivation was studied at different physiological, biochemical, and molecular levels.

## Materials and Methods

### Plant Material and Growth Conditions

The hydroponic experiments were conducted in a greenhouse equipped with high-pressure sodium lights (60% humidity, 16 h days at 21°C, and 8 h night at 18°C). Rapeseed seeds (cv Trezzor) were germinated for 20 days on vermiculite and were then transplanted to 7 L tray with 0.5x Hoagland solution, each containing 12 plants. Hydroponics solution was buffered to pH 5.9 and renewed two times a week with full Hoagland solution [2 mM Ca(NO_3_)_2_, 1 mM K_2_SO_4_, 1 mM MgSO_4_, 0.5 mM NH_4_H_2_PO_4_, 0.5 mM CaCl_2_, 10 μM H_3_BO_3_, 2.5 μM MnSO_4_, 0.5 μM ZnSO_4_, 0.2 μM CuSO_4_, 0.01 μM (NH_4_)_6_Mo_7_O_24_, and 100 μM EDTA,2NaFe] with continuous aeration. K deprivation was started 14 days after transplanting in K-sufficient condition by removing K_2_SO_4_ from the medium. Samples were harvested at 24 h, 3, 7, and 14 days after K deprivation (each time, six plants were harvested). For the last harvest, the total fresh weight of the shoots and the roots was determined.

### RNA Extraction and Gene Expression Analysis

The root and leaf samples (100 and 70 mg, respectively) of rapeseed plants harvested for each time point (24 h, 3, 7, and 14 days) were ground to a fine powder in the presence of liquid N, and the total RNA was extracted using a Nucleospin^®^8 RNA kit (Macherey-Nagel, Düren, Germany) following the protocol of the manufacturer. The quality and yield of all RNA samples were analyzed and checked in a 4200 Tapestation (Agilent Technologies, Santa Clara, CA, United States), followed by DNase treatment and complementary DNA (cDNA) synthesis from 1 μg RNA using an iScript genomic DNA (gDNA) clear cDNA synthesis kit (Bio-Rad, Hercules, CA, United States). Quantitative RT-PCR (qPCR) analysis was performed in a total volume of 10 μl using Universal SYBR Green Supermix (Bio-Rad, Hercules, CA, United States) in a RT PCR Detection System (Bio-Rad, Hercules, CA, United States). The qPCR reactions were performed in technical triplicates using independent cDNA reactions for each biological replicate and 300 nM of gene-specific primer pairs. Specific primers for all candidate genes were designed using Primer3 software (version 0.4.0) and are listed in Supplementary Table 1. The thermal cycler protocol included 98°C for 3 min, 40 cycles of 98°C for 15 s, 60°C for 30 s, 72°C for 15 s, and a final 3-min extension at 72°C. The expression of all the candidate genes was normalized against four rapeseed reference genes, namely *BnaEf1alpha*, *BnaACT7*, *BnaTIP41*, and *BnaPP2A*. The selection of four reference genes was based on the different published literatures comparing the reference gene stability in stress conditions in Brassica species (Wang et al., 2014; Ma et al., 2020). We analyzed the four reference genes in both the roots and the leaves of the experimental samples and used the inbuilt Reference Gene Selection tool provided with CFX Maestro software version 1.0 (Bio-Rad, Hercules, CA, United States) to evaluate the stability of these reference genes in all the tested samples. Based on the stability values of these reference genes in experimental tissue samples, we chose only the ideal reference genes in each tissue for normalization of expression. Therefore, the expression in the shoots were normalized to the stable *BnaACT7* and *BnaEf1alpha* reference genes, while in the roots, the expression values were normalized in all four reference genes (*BnaEf1alpha, BnaACT7, BnaTIP41, and BnaPP2A*). Normalized expression of each sample for selected candidate genes were calculated using CFX Maestro Software Version 1.0 (Bio-Rad, Hercules, CA, United States). Statistics were performed for each time point independently.

### Determination of Mineral Elements

Elemental analysis was performed using inductively coupled plasma-optical emission spectrometry (ICP-OES, 5110 VDV, Agilent, CA, United States) with prior microwave acid sample digestion (Multiwave Pro, Anton Paar, Les Ulis, France) [8 ml of concentrated HNO_3_, 2 ml of H_2_O_2_, and 15 ml of Milli-Q water for 100 mg dry weight (DW)]. The quantification of each element was carried out with an external standard calibration curve. Analysis of N was realized using an elemental FLASH 2000 CHNS analyzer (Thermo Fisher Scientific, Waltham, MA, United States) according to the instructions of the manufacturer from 2.5 mg of homogenized and lyophilized plant material. NH_4_^+^, NO_3_, and sulfate (SO_4_^2–^) were extracted and analyzed according to Réthoré et al. (2020) using high pressure ion chromatography with a conductivity detector (Dionex ICS5000 +, Thermo Fisher Scientific, Villebon-sur- Yvette, France).

### Determination of Primary Metabolites

For amino acid determination, 10 mg of lyophilized dry matter was extracted with a solution containing 400 μl of MeOH and 0.250 nmol/μl of Norvaline, which was used as the internal standard (Sigma Aldrich, St. Louis, MO, United States). The extract was stirred for 15 min, and it was then re-suspended with 200 μl of chloroform (agitation for 5 min) and 400 μl of double-distilled water (ddH_2_O). After centrifugation (12,000 rpm, 10°C, 5 min), the supernatant was recovered, evaporated, and dissolved in 100 μl of ddH_2_O. Derivatization was performed using an Ultra Derivatization Kit AccQ tag (Waters Corp, Milford, MA, United States), following the protocol of the manufacturer (Waters Corp, Milford, MA, United States). The amino acid profile was determined by ultra-performance liquid chromatography coupled with a photodiode array detector (UPLC/PDA) H-Class system (Waters Corp, Milford, MA, United States) with an ethylene bridge hybrid (BEH) C18 100 × 2.1 mm column (pore size: 1.7 μm). Since methionine and O-acetylserine (OAS) could not be determined using this method, they were extracted and analyzed according to the method described by Ali et al. (2018).

Soluble sugar determination was undertaken according to the method described by Kim et al. (2013). Ten mg of lyophilized shoot material was homogenized in liquid nitrogen, dissolved in 0.75 ml of 80% (v/v) ethanol, and incubated at 80°C for 30 min. Crude extracts were decanted for 15 min, centrifuged at 14,000 rpm for 10 min at 4°C, and concentrated in a Speed Vac concentrator (Thermo Fisher Scientific, Waltham, MA, United States) at 45°C for 180 min. The pellet was resuspended in 0.75 ml of deionized water and incubated at 80°C for 30 min. After centrifugation, the second supernatant was added to the first, concentrated, and resuspended in 0.5 ml of ddH_2_O. Hexokinase (HK), phosphoglucoisomerase (PGI), and beta-fructosidase were added successively to measure glucose (Glc), fructose (Fru), and sucrose (Suc), which were determined by spectrophotometry at 340 nm (SpectraMax i3x, Molecular Devices, San Jose, CA, United States).

Polyamine extraction was achieved using 20 mg of frozen ground leaves or roots that were weighed in a 2 ml Eppendorf tube (Eppendorf, Hamburg, Germany). Extraction was carried out by adding 1 ml of a solution of 70% H_2_O/29% MeOH/1.0% formic acid (v/v/v) at −20°C. Samples were vortexed then centrifuged at 4°C (16,000 rpm), and the supernatant was transferred to a LC/MS vial for analysis. Polyamines were analyzed by a UHPLC–MS/MS system. The separation and detection were achieved using a Nexera X2 UHPLC System (Shimadzu, Kyoto, Japan) coupled to a QTrap 6500 + mass spectrometer (Sciex, Concord, ON, Canada) equipped with an IonDrive^TM^ turbo V electrospray source. The separation was carried out by injecting a 2 μl of sample into a Synergi Hydro-RP column (100 × 2.0 mm, 2.5 μm, Phenomenex, United States) at a flow rate of 0.7 ml/min, and the column temperature was maintained at 40°C. The mobile phases were composed of solvent A Milli-Q water (18 Ω, Millipore, United States) containing 0.1% perfluorooctanoic acid (PFOA) (Merck KGAA, Darmstadt, Germany), and solvent B acetonitrile LCMS grade (Fisher Optima, Fisher, United Kingdom). The gradient elution started with 10% B, 0.0–2.0 min 40% B, 2.0–3.5 min 100% B, 3.5–5.0 min 100% B, 5.0–5.5 min 10% B, and 5.5–7.5 min 10% B. The MS acquisition was performed in a scheduled MRM mode in a positive mode with the following parameters: Ion spray voltage 5,500 V; source temperature 600°C; Curtain gas 35 psi; nebulizer gas 50 psi; heater gas 60 psi; collision gas medium; entrance potential + 10 V; MRM detection window 60 s; target scan time 0.25 s.

### Determination of Phytohormones

Phytohormones (Abscisic acid, ABA; Phaseic acid, PA; Dihydrophaseic acid, DPA; Salicylic acid, SA; 12-oxo-phytodienoic acid, OPDA; Jasmonic acid, JA; Jasmonoyl-Isoleucine, JA-Ile; 1-amino-1-cyclopropane-carboxylic acid, ACC; Indole-3-acetic acid, IAA) standards were purchased from Sigma (Lyon, France) and OlchemIn (Olomouc, Czech Republic). Stable isotope-labeled internal standards, d6-ABA, d3-DPA, d3-PA, d4-SA, d5-JA, d4-ACC, and d5-IAA were ordered from National Research Council of Canada (Ottawa, Canada) and OlchemIn. Then, 10 mg of the frozen shoots and roots were extracted with 70% of methanol, 29% of H_2_O, 1% of formic acid containing isotope-labeled internal standards, and centrifuged at 12,600 rpm to collect the supernatant. After evaporation (SPE Dry 96, Biotage, Uppsala, Sweden), the extract was resuspended in 2% of formic acid solution and purified using a SPE ABN (Solid Phase Extraction, Acidic, Basic and Neutral Analytes) express plate of 30 mg/ml (Biotage, Uppsala, Sweden). The phytohormones were eluted with methanol, and the samples were evaporated and resuspended in 200 μl of 0.1% formic acid solution before injected into the system. The separation and detection were achieved using a Nexera X2 UHPLC system coupled to a QTrap 6,500 + mass spectrometer equipped with an IonDrive turbo V electrospray source. Phytohormone separation was carried out by injecting a 2 μl sample into a Kinetex Evo C18 core–shell column (100 × 2.1 mm, 2.6 μm, Phenomenex, United States) at a flow rate of 0.7 ml/min, and the column temperature was maintained at 40°C. The mobile phases were composed of solvent A Milli-Q water containing 0.1% of formic acid (LCMS grade, Fluka analytics, Germany), and solvent B acetonitrile LCMS grade containing 0.1% of formic acid. The gradient elution started with 1% of B, 0.0–5.0 min 60% of B, 5.0–5.5 min 100% of B, 5.5–7.0 min 100% of B, 7.0–7.5 min 1% of B, and 7.5–9.5 min 1% of B. The analysis was performed in a scheduled MRM mode in positive and negative modes simultaneously with a polarity switching of 5 ms: Ion spray voltage 5,500 V in the positive mode and −4500 V in the negative mode; Source temperature 600°C; curtain gas 35 psi; nebulizer gas 50 psi; heater gas 60 psi; collision gas medium; entrance potential ± 10 V; MRM detection window 30 s; target scan time 0.075 s. An external calibration curve was established for each phytohormone. A quality control sample containing all external and internal standards was injected to every 10 samples to access the system stability. The calculated concentration was corrected by the internal standard recovery rate.

### Statistical Analysis

Data are represented as boxplots representing all datapoints, or bar plots indicating mean ± standard error of the mean (SEM) for *n* = 6. *T*-test (R software) was employed to analyze the data at each time point independently and graphs were marked by asterisks when significantly different (^∗^*p* < 0.05; ^∗∗^*p* < 0.01, and ^∗∗∗^*p* < 0.001, *n* = 6). Boxplot graphs were created using GraphPad Prism 9.0.1 software (GraphPad Software, San Diego, CA, United States).

## Results

### Effect of K Deprivation on Plant Growth, Mineral Status, and the Expression of the Genes Involved in K, S, and N Transport

**FIGURE 1:**
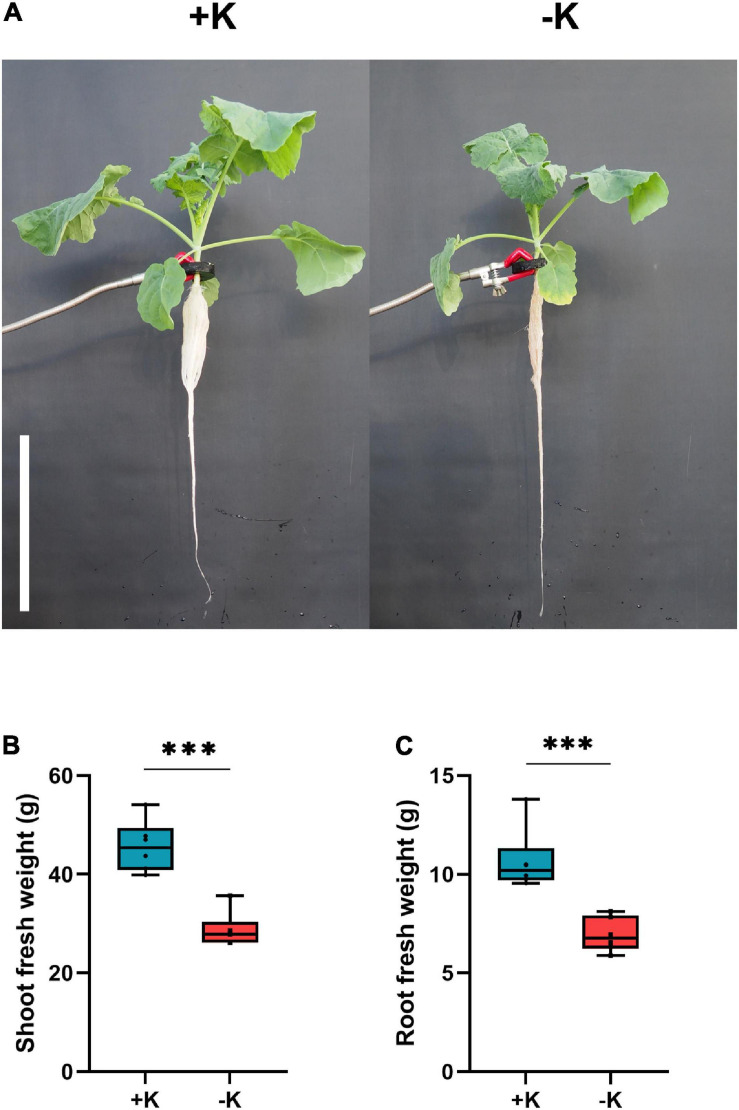
Influence of K deficiency on rapeseed growth and development. **(A)** Representative picture of K-sufficient (+ K) and K-starved plants (–K); **(B)** shoot fresh weight and **(C)** root fresh weight. Plants were grown in a hydroponic culture under either –K (0 mM) or + K (2 mM). Roots and shoots were harvested after 14 days of K deprivation. Asterisks denote the significant level of the difference according to *t*-test (^∗^*p* < 0.05, ^∗∗^*p* < 0.01, and ^∗∗∗^*p* < 0.001, *n* = 6). The scale bar represents 25 cm.

The effect of K deprivation on rapeseed biomass was evaluated 14 days after the beginning of stress application (Figure 1A). A significant decrease in the biomass of both the roots and the shoots was observed (Figures 1B,C). Compared to the ample K condition, the root and shoot fresh weights decreased by 37 and 35%, respectively.

**FIGURE 2:**
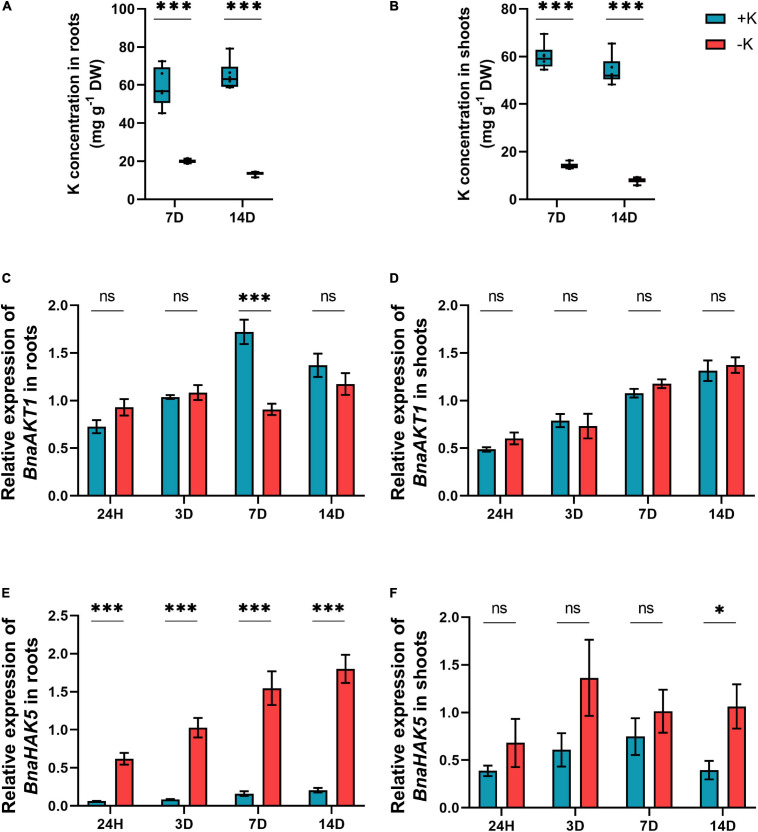
Influence of K deficiency on K concentration and the expression of the genes involved in K transport in rapeseed plants. **(A)** K concentration in roots; **(B)** K concentration in shoots; **(C)** relative expression of *BnaAKT1* in roots; **(D)** relative expression of *BnaAKT1* in shoots; **(E)** relative expression of *BnaHAK5* in roots and **(F)** relative expression of *BnaHAK5* in shoots. Plants were grown in a hydroponic culture under either –K (0 mm) or + K (2 mm). Roots and shoots were harvested after 24 h, 3, 7, or 14 days of K deprivation. Bars indicate mean ± standard error of the mean (SEM) (*n* = 6). Asterisks denote the significant level of the difference according to *t*-test (^∗^*p* < 0.05, ^∗∗^*p* < 0.01, and ^∗∗∗^*p* < 0.001, *n* = 6). Ns denotes non-significant differences.

In the next approach, the influence of 7 and 14 days of K deprivation on mineral nutrition status was assessed both in the roots and shoots using ICP-OES as well as elemental carbon, hydrogen, and nitrogen (CHN) analyzer. As expected, after 7 days of K deprivation, the K levels sharply decreased in both the plant parts (−66% in the roots and −76% in the shoots) (Figures 2A,B). This decrease was even more pronounced when the duration of K deficiency was prolonged for 2 weeks (Figures 2A,B). In order to better characterize the response of *B. napus* to K deficiency, the expression level of K transporters was also monitored in both the roots and the shoots after short-term (24 h or 3 days) or long-term K deprivation (7 and 14 days). The expression of the low-affinity transporter *BnaAKT1* was not significantly affected by low K treatment in the shoots and was only significantly decreased after 7 days in the roots (Figures 2C,D). In addition, and compared to ample K condition, the transcript level of high-affinity K transporter, *BnaHAK5* which mediates the K uptake under low K, was significantly induced in the roots at all-time points under K deprivation (Figure 2E). This trend was not observed in the shoots and the *BnaHAK5* expression was induced only in response to long-term K deficiency (14 days) (Figure 2F).

**FIGURE 3:**
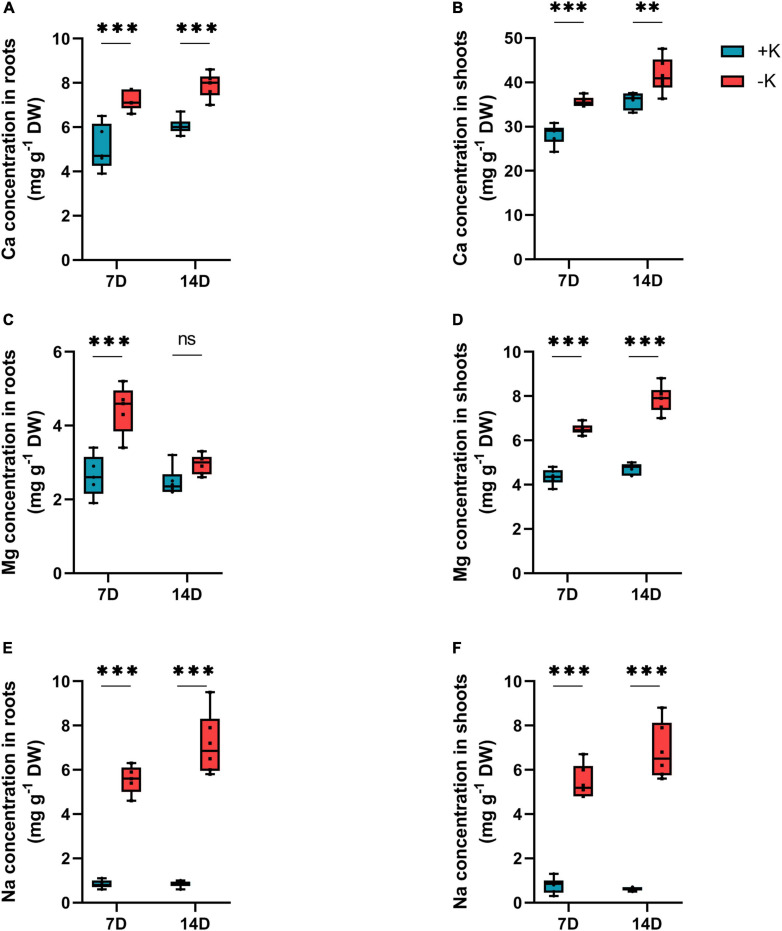
Influence of K deficiency on mineral nutrition status in rapeseed plants. **(A)** Ca concentration in roots; **(B)** Ca concentration in shoots; **(C)** Mg concentration in roots; **(D)** Mg concentration in shoots; **(E)** Na concentration in roots, and **(F)** Na concentration in shoots. Plants were grown in a hydroponic culture under either –K (0 mM) or + K (2 mM). The roots and shoots were harvested after 7 or 14 days of K deprivation. Asterisks denote the significant level of the difference according to *t*-test (^∗^*p* < 0.05; ^∗∗^*p* < 0.01, and ^∗∗∗^*p* < 0.001, *n* = 6).

Determining the levels of other cations showed that K deficiency induced a significant accumulation of cations, Mg^2+^, Ca^2+^, and Na^+^ in both the roots and shoots (Figures 3A–F). The higher accumulation of these cations under K deficiency was expected, but it was more pronounced in the case of Na^+^ (Figures 3E,F).

**FIGURE 4:**
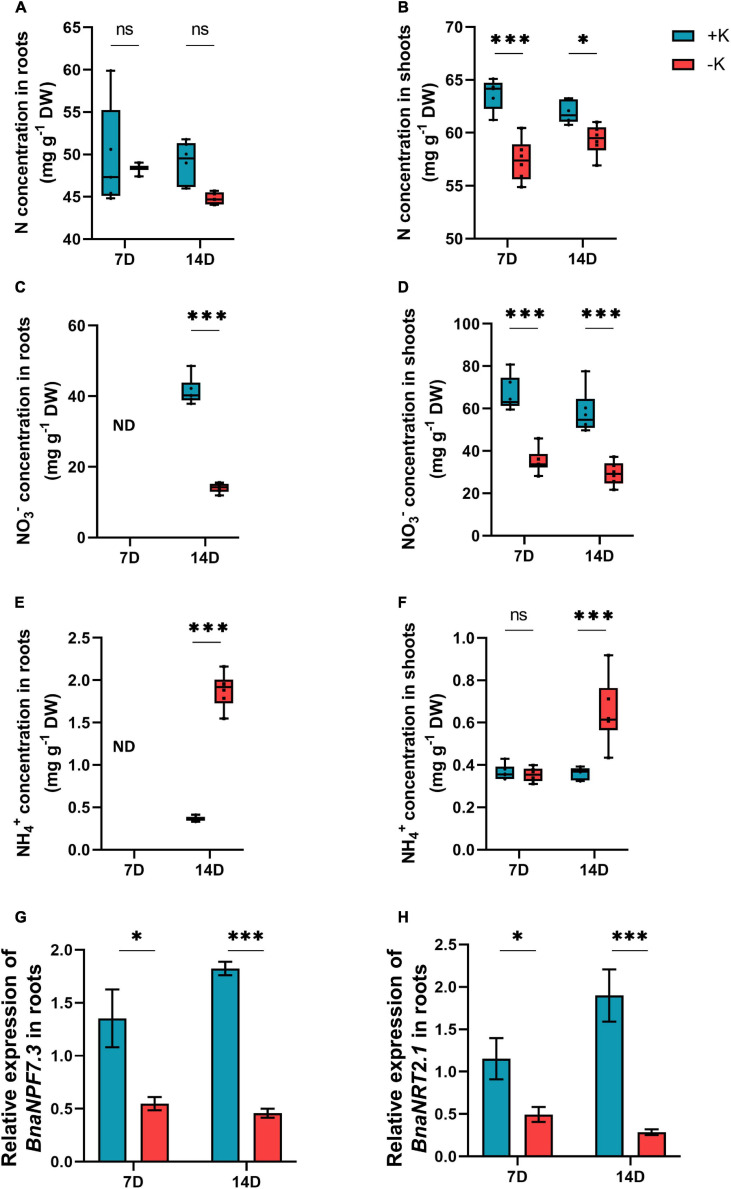
Influence of K deficiency on N concentration and the expression of nitrate transporter in rapeseed. **(A)** N concentration in roots; **(B)** N concentration in shoots; **(C)** NO_3_^–^ concentration in roots; **(D)** NO_3_^–^ concentration in shoots; **(E)** NH_4_^+^ concentration in roots; **(F)** NH_4_^+^ concentration in shoots; **(G)** relative expression of *BnaNPF7.3* in roots and **(H)** relative expression of *BnaNRT2.1* in roots. Plants were grown in a hydroponic culture under either –K (0 mM) or + K (2 mM). The roots and shoots were harvested after 7 or 14 days of K deprivation. Bars indicate mean ± standard error of the mean (SEM) (*n* = 6). Asterisks denote the significant level of the difference according to *t*-test (^∗^*p* < 0.05, ^∗∗^*p* < 0.01, and ^∗∗∗^*p* < 0.001, *n* = 6). Ns denotes non-significant differences.

We also observed a decline in the levels of N by the application of K deficiency; however, this reduction was only statistically significant in the shoots (Figures 4A,B). Looking at the concentrations of different forms of N, we observed that the NO_3_^–^ levels decreased in the roots after 14 days and in the shoots after 7 and 14 days of low K supply (Figures 4C,D) while the levels of NH_4_^+^ significantly increased in the roots after 14 days of K deprivation (Figure 4E). Notably, the level of NH_4_^+^ did not change in the shoots after 7 days of K deficiency and significantly increased at day 14 (Figure 4F). The lower concentrations of N under K deficiency encouraged us to further investigate if K stress modulates the genes involved in N transport in *B. napus*. In fact, the expression levels of the high-affinity of NO_3_^–^ transporter, *BnaNRT2.1* involved in NO_3_^–^uptake were declined in the roots irrespective of the time of application of K deficiency (Figure 4H). The same pattern was also observed for the expression pattern of the low-affinity NO_3_^–^ transporter, *BnaNPF7.3* which is involved in the xylem loading of NO_3_^–^(Figure 4G).

**FIGURE 5:**
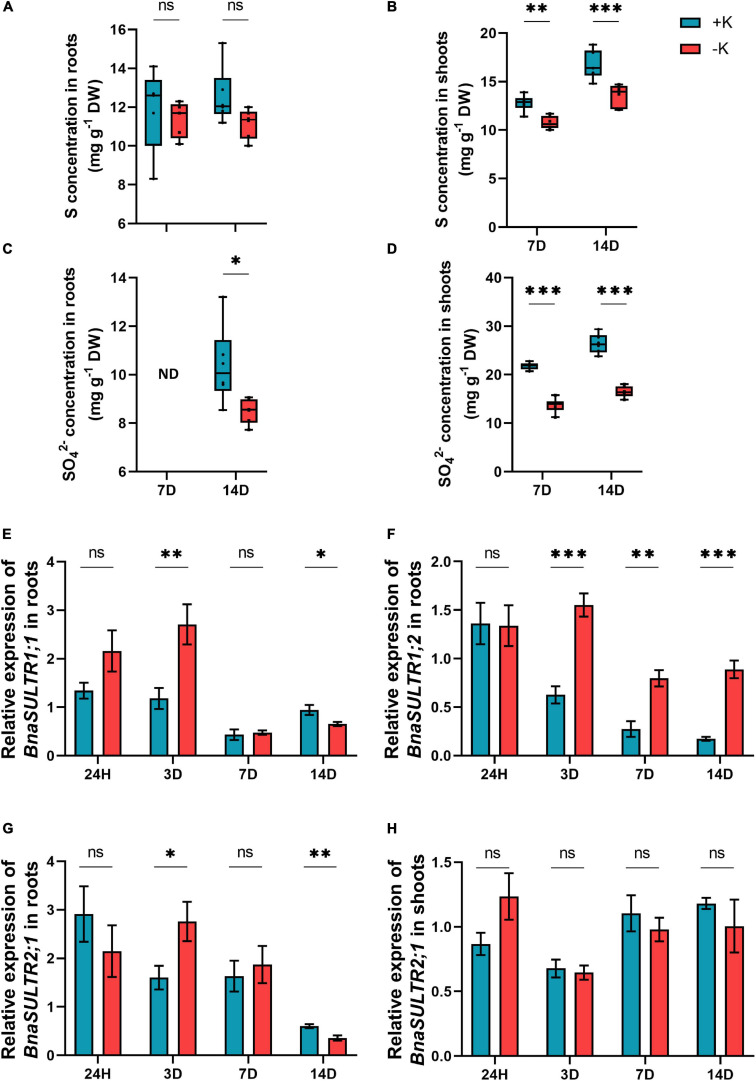
Influence of K deficiency on S status and the genes involved in S transport in rapeseed plants. **(A)** S concentration in roots; **(B)** S concentration in shoots; **(C)** SO_4_^2–^ concentration in roots; **(D)** SO_4_^2–^ concentration in shoots; **(E)** relative expression of *BnaSULTR1;1* in roots; **(F)** relative expression of *BnaSULTR1;2* in roots; **(G)** relative expression of *BnaSULTR2;1* in roots and **(H)** relative expression of *BnaSULTR2;1* in shoots. Plants were grown in a hydroponic culture under either –K (0 mM) or + K (2 mM). Roots and shoots were harvested after 24 h, 3, 7, or 14 days of K deprivation. Bars indicate mean ± standard error of the mean (SEM) (*n* = 6). Asterisks denote the significant level of the difference according to *t*-test (^∗^*p* < 0.05, ^∗∗^*p* < 0.01, and ^∗∗∗^*p* < 0.001, *n* = 6). Ns denotes non-significant differences.

Like N, the levels of S also did not change in the roots but was significantly decreased in the shoots after both 7 and 14 days of low K stress (Figures 5A,B). This is held true for the levels of SO_4_^2–^ both in the roots and shoots (Figures 5C,D). We then examined the expression level of high-affinity S transporter, *BnaSULTR1;1* which was induced under short-term K deficiency in the roots and was even statistically significant already after 3 days of low K stress (Figure 5E). The expression pattern of this gene then decreased upon 7 days of K deficiency and was identical in both low and ample K condition (Figure 5E). Compared to 7 days K stress, the expression levels of *BnaSULTR1;1* slightly increased after 14 days, but its expression was significantly lower compared to + K plants (Figure 5E). The same pattern was observed for another high affinity S transporter, *BnaSULTR1;2* which was significantly induced after 3 days under low K in comparison to ample K condition (Figure 5F). Moreover, the expression of *BnaSULTR1;2* remained induced after 7- and 14-days K starvation. This induction was less compared to short-term K deficiency (3 days) (Figure 5F). The expression level of the low affinity S transporter, *BnaSULTR2;1*, which is involved in long-distance S transport from the root to the shoot (Lewandowska and Sirko, 2008; Takahashi et al., 2011), was also evaluated in both the roots and the shoots in response to K deficiency. Except for a significant increase after 3 days and a decrease at 14 days in the roots in response to low K, there was no significant change in the expression level of *BnaSULTR2;1* in the roots and the shoots (Figures 5G,H). Altogether, these data revealed that *B. napus* is very sensitive to K deficiency and modulated the expression pattern of the genes involved in macronutrient transport, such as N and S when K is depleted from the medium.

### Effect of K Deprivation on Primary Metabolites and ABA Level in Rapeseed

**FIGURE 6:**
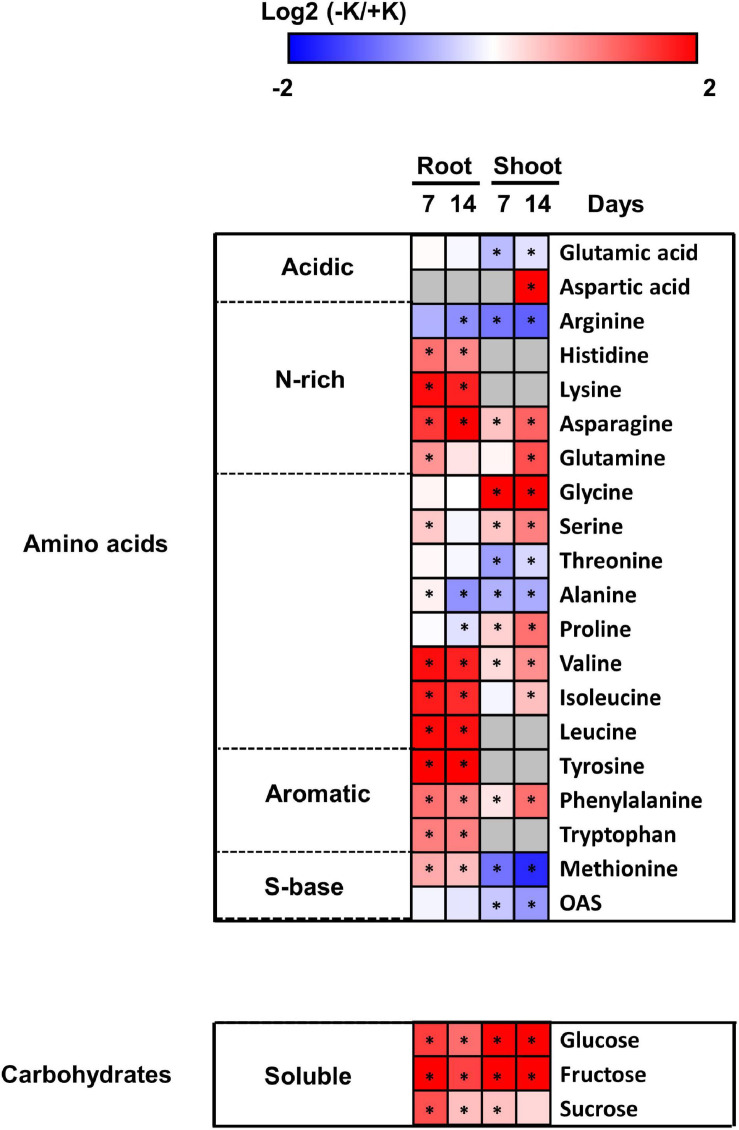
Heatmap representation of primary metabolites in rapeseed plants exposed to K deficiency. The calculation was based on the log2 values of the ratio between –K/^+^ K for each of the metabolites at 7 or 14 day. Asterisks denote significant differences according to *t*-test (*p* < 0.05).

The effect of K deprivation on *B. napus* metabolism was also evaluated by quantifying the concentration of soluble sugars, amino acids, and polyamines in both the roots and the shoots after 7 or 14 days of K deprivation (Supplementary Table 2). Most of the amino acids varied in a similar pattern in *B. napus* in both parts (Figure 6). We observed a significant increase in N-rich amino acids. Indeed, asparagine was accumulated in both the roots and the shoots at 7 and 14 days of K starvation, while the level of glutamine increased at 7 days in the roots and at 14 days in the shoots. The levels of both histidine and lysine were increased in the roots at both time points. The concentration of aromatic amino acids (tyrosine, phenylalanine, and tryptophan) was significantly increased in both the roots and shoots, while phenylalanine level was significantly increased in the shoots. On the contrary, the concentration of arginine decreased in both roots and shoots at all-time points of K deficiency. In addition, we observed a significant decrease in the levels of glutamic acid after 7 and 14 days and an increase in aspartic acid in the shoots after 14 days. In the roots, aspartic acid was only quantifiable under low K supply (Figure 6). Notably, the concentration of proline, which is known to provide better stress tolerance in plants, significantly increased in the shoots after 14 days of K deficiency (Figure 6). The levels of both Glycine (Gly) and serine (Ser) were also significantly accumulated in the shoots of K-deficient plants after 7 and 14 days of stress.

Then we measured the concentrations of soluble sugars in response to K deprivation. In general, the levels of glucose, fructose, and sucrose were strongly increased in both the roots and shoots either after 7 or after 14 days of the K stress. As expected, due to the accumulation of sugar in the shoots under long-term K stress, the concentrations of Glu and Fru was relatively lower in the roots compared to the shoots (Figure 6). It is worthy to note that unlike these two soluble sugars, the accumulation of sucrose in the shoots was not statistically significant after 14 days of low K supply (Figure 6).

**FIGURE 7:**
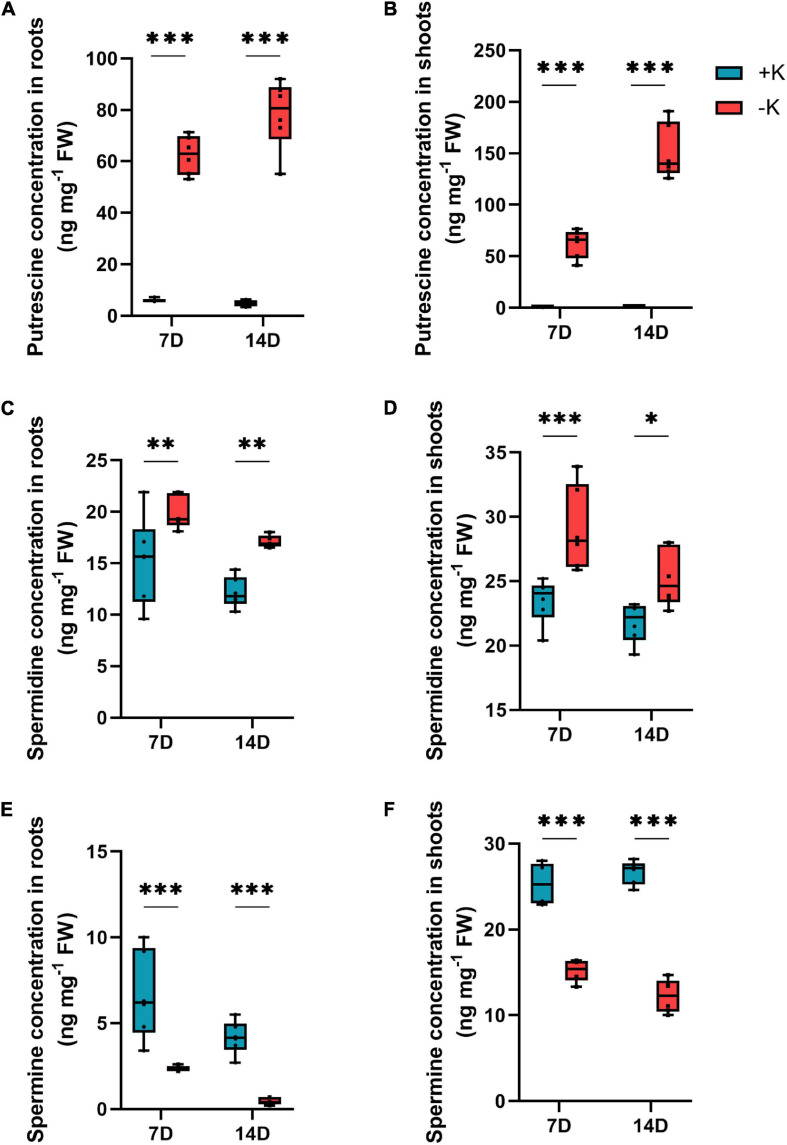
Influence of K deficiency on the concentration of polyamines in rapeseed plants. **(A)** Putrescine (Put) concentration in roots; **(B)** Put concentration in shoots; **(C)** spermidine concentration in roots; **(D)** spermidine concentration in shoots; **(E)** spermine concentration in roots and **(F)** spermine concentration in shoots. Plants were grown in a hydroponic culture under either –K (0 mM) or + K (2 mM). The roots and shoots were harvested after 7 or 14 days of K deprivation. Asterisks denote the significant level of the difference according to *t*-test (^∗^*p* < 0.05, ^∗∗^*p* < 0.01, and ^∗∗∗^*p* < 0.001, *n* = 6).

Further, we evaluated the effect of K deficiency on the level of polyamines. Unlike spermine, which was significantly reduced in both the roots and the shoots at low K supply (Figures 7E,F), the levels of spermidine and Put increased in both the parts (Figures 7A–D). Among these two polyamines, Put was found to be one of the most accumulated metabolites under K deficiency in *B. napus*. In the roots, the Put level significantly increased by 11-fold after 7 days and 16-fold after 14 days. However, this increase was less pronounced in the roots compared to the shoots. The Put level increased in the shoots by 72-fold after 7 days and by 112-fold after 14 days of K withdrawal. Overall, these results demonstrate that K deficiency induced the changes in the levels of amino acids and increased the polyamines levels in *B. napus* in response to K starvation.

**FIGURE 8:**
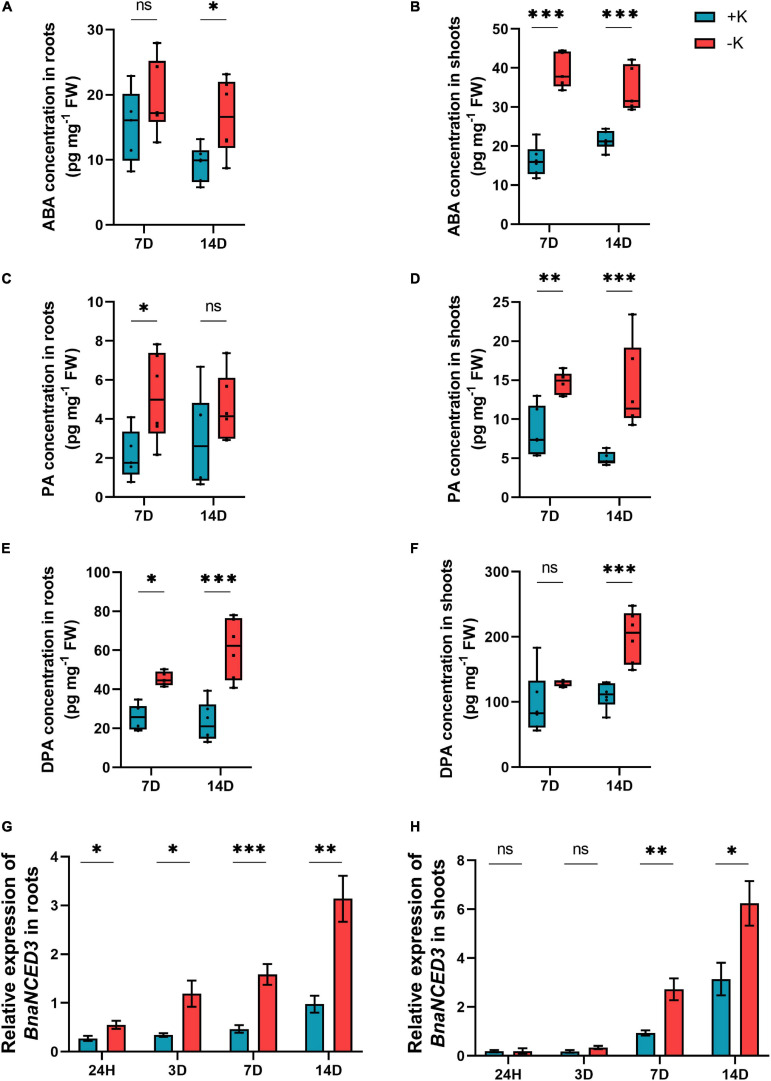
Influence of K deficiency on ABA level and the key genes involved in ABA biosynthetic pathway in rapeseed. **(A)** ABA concentration in roots; **(B)** ABA concentration in shoots; **(C)** phaseic acid (PA) concentration in roots; **(D)** PA concentration in shoots; **(E)** diphaseic acid (DPA) concentration in roots; **(F)** DPA concentration in shoots; **(G)** relative expression of *BnaNCED3* in roots and **(H)** relative expression of *BnaNCED3* in shoots. Plants were grown in a hydroponic culture under either –K (0 mM) or + K (2 mM). The roots and shoots were harvested after 24 h, 3, 7, or 14 days of K deprivation. Bars indicate mean ± standard error of the mean (SEM) (*n* = 6). Asterisks denote the significant level of the difference according to *t*-test (^∗^*p* < 0.05, ^∗∗^*p* < 0.01, and ^∗∗∗^*p* < 0.001, *n* = 6). Ns denotes non-significant differences.

Since hormones play a major role in the responses to nutritional stresses (Martínez-Andújar et al., 2016) and cross-talk between ABA and K deficiency has been already established (Mansfield and Jones, 1971; Schraut et al., 2005), the concentrations of ABA and its derivatives were determined in both the roots and shoots in response to K deprivation. In the roots, the concentration of ABA was significantly increased after 14 days of K deficiency compared to the ample K condition, whereas in the shoots, the increase was significant at both 7 and 14 days after K deprivation (Figures 8A,B). Looking at the concentrations of ABA degradation metabolites, phaseic acid (PA) was also significantly accumulated after 7 days, while its levels increased in the shoots at 7 and 14 days of K stress (Figures 8C,D). Moreover, the concentration of diphaseic acid (DPA) was significantly increased after 7 and 14 days of low K supply in the roots and after 14 days in the shoots (Figures 8E,F). We then examined the expression pattern of *BnaNCED3*, a key gene involved in ABA biosynthetic pathway (Xu and Cai, 2017). Interestingly, we found an induction in the expression levels of *BnaNCED3* which was associated with the higher level of ABA (Figures 8G,H). Indeed, the K deficiency significantly induced the transcript levels of this gene in the roots at the beginning of K stress (1 day) which was then sharply induced when K deficiency prolonged up to 14 days. In the shoot, the expression of *BnaNCED3* was also significantly induced after 7 and 14 days of K starvation. Altogether, these results revealed a clear effect of K deficiency on ABA homeostasis in *B. napus* plants exposed to K deficiency. We did not observe any particular changes in the level of other phytohormones.

## Discussion

Despite a profound investigation on the role of K for plant growth and development in other crop species, less attention has been given to its role in *B. napus* plant compared to N and S nutrition. Here, we tackled the question of how the *B. napus* responds to K starvation and what are the overall changes in terms of metabolism when encountering K deprivation. Compared to normal K level, the results indicated the different physiological, biochemical, and molecular responses either under short or long K deficiency.

### K Deficiency Reduced the Growth and Imposed Nutritional Imbalances in *B. napus*

*Brassica napus* demands higher quantity of nutrition of minerals for the proper growth and development compared to the other crops (Maillard et al., 2016). It is well-documented that any individual deficiencies can disturb the nutritional balance and thus poses the yield penalty (Marschner, 2012). K is one of the essential macronutrients which plays a pivotal function in enzyme activation, osmotic adjustment, turgor generation, cell expansion, regulation of membrane electric potential, and pH homeostasis (Marschner, 2012; Ragel et al., 2019). It is the most abundant cation in plant cells, comprising up to 10% of plant dry weight; hence, the growth of plants declines rapidly under low supply of K (Hermans et al., 2006; Schachtman, 2015). The decrease in both the root and the shoot biomass under K starvation has already been shown in different crop plants, such as apple (Chang et al., 2014), wheat (Damon and Rengel, 2007), and soybean (Singh and Reddy, 2017). In the present work and in line with the above findings, 2-weeks K deficiency significantly diminished the growth in *B. napus*, indicating the pivotal role of K in the growth and development of rapeseed plants.

The uptake of mineral nutrients is coordinated by a complex network and is tightly regulated (Marschner, 2012). The lack of one nutrient influences the uptake of the other nutrients. In addition, mineral nutrients interact either synergistically or antagonistically at different levels. Some of these interactions have already been described extensively. For instance, the inter-relation between N and S has been shown in numerous studies (Koprivova et al., 2000; Hesse et al., 2004; Coleto et al., 2017). In another study in rapeseed, Maillard et al. (2016) showed that the K deficiency induced an increase in the levels of Na, Mg, and Ca. A similar effect was also shown in barley plants exposed to K deficiency (Hosseini et al., 2016). In the present work, the K levels drastically decreased, and the depletion of K was even more pronounced when the duration of K deficiency was prolonged to 14 days. In addition, the K deprivation imposed a significant increase in the levels of Ca, Mg, and Na both in the roots and shoots. This effect was expected as K is replaced at a varying degree in the vacuole by other cations (Marschner, 2012) and was shown already in several studies on oil palm and sunflower (Cui et al., 2019a, b). In general, under K deprivation, a considerable increase in the concentrations of the cations, such as Mg and Ca provide the positive charge; thus, the K-starved plants demand an additional negative charge to balance the electric charge which is mainly provided *via* accumulation of organic or amino acids (Armengaud et al., 2009; Cui et al., 2020b). Apparently, this response was not true in K-starved *B. napus* plants since the total calculated positive charge was not remarkable. However, Ca contribution seems more pronounced compared to the other cations (Supplementary Figure 1). Cui et al. (2021) showed that in the sunflower, Ca plays a significant role to mitigate the response of K deficiency, such as lower photosynthesis and N assimilation (Cui et al., 2021).

Conversely, the level of N and S in the shoots declined under both short- and long-term K deficiency which was in agreement with the finding by Maillard et al. (2016), and highlight the fact that K is crucial for the uptake of both N and S in rapeseed. The interaction between N and K was shown previously. Indeed, K deficiency has been shown to decrease the assimilation of N. K is transported together with NO_3_^–^ in the xylem, and K stimulates the uptake of NO_3_^–^ in the roots and its translocation to the shoots (Dong et al., 2004; Drechsler et al., 2015; Hu et al., 2016). Furthermore, K has a similar charge, size, and hydration energy, and characteristics like NH_4_^+^; therefore, K competes with NH_4_^+^ (Hoopen et al., 2010). Notably, in this study, in general, the concentration of NO_3_^–^ was extremely higher compared to NH_4_^+^, but the uptake of NH_4_^+^ increased in the roots compared to NO_3_^–^ whose level declined both in the roots and the shoots upon K starvation. This indicates that the rapeseed plants uptake more NH_4_^+^ under K deficiency. This higher uptake could be in favor of a higher GS/GOGAT/GDH activity under K deprivation, as observed in the roots of *Arabidopsis* (Armengaud et al., 2009). Altogether, our results showed that the K deficiency changes the nutritional balances in rapeseed which was accompanied with a decrease in the levels of S and N and a simultaneous increase of positively charged cations, such as Ca and Mg.

### Potassium Deficiency Changes the Expression of the Genes Involved in K, S, and N Transport

The availability of minerals in the soil is dynamic and heterogeneous. It is already shown that, *AKT1* is expressed highly in the roots, and considered as a low-affinity K transporter which mediates K uptake when external concentrations are between sub-millimolar and millimolar ranges (Cheong et al., 2007; Luan et al., 2016). *HAK5* is also another high-affinity K transporter which is responsible for K uptake when the external level is lower than 10 μm (Hong et al., 2013; Meng et al., 2016). It is worthy to note that *AKT1* plays a dominant role in K uptake when a higher concentration of NH_4_^+^ is present (Hoopen et al., 2010). However, *HAK5* transcription is shown to be strongly upregulated under low K starvation (Hong et al., 2013). Monitoring the expression of these two genes showed that the transcript level of *BnaHAK5* was induced from the onset of application of K deficiency and its induction was even more pronounced when K deficiency was prolonged for 2 weeks. This indicated that in rapeseed, *BnaHAK5* is the dominant transporter which responds to K starvation.

The lower concentration of N and S induced by K starvation also encouraged us to examine the expression of the genes involved in their transport. The two S transporters, *SULTR1;1*, and *SULTR1;2* which are mainly expressed in the roots are responsible for S uptake within the plants (Gigolashvili and Kopriva, 2014). While *SULTR1;1* has been shown to function during S starvation (Yoshimoto et al., 2002), *SULTR1;2* is the more prominent transporter under normal S supply (Rouached et al., 2008), and has been proposed to act as a sensor for S status of plants (Zhang et al., 2014). The expression of both genes was shown to be increased in rapeseed plants exposed to S deficiency (Sorin et al., 2015; Maillard et al., 2016). Consistent with these observations, the transcript levels of these genes were upregulated under K starvation highlighting the fact that the homeostasis of S status in the rapeseed was disturbed by the lack of K. One might consider that it could be due to the halved S content after removing K_2_SO_4_ from the nutrient solution to induce K deficiency. Hence, this was further examined by monitoring the expression level of O-acetylserine (thiol)lyase (*BnaOASTL*) (Murad et al., 2021) which did not induce under K deprivation (Supplementary Figure 2) and there were also no significant changes in the concentration of OAS, which is one of the best S deficiency markers (Kaur et al., 2011). In addition, the rapeseed needs a high amount of N to maintain normal growth and development and is extremely susceptible to N deficiency. The corresponding N transporters were also identified in rapeseed (Zhang et al., 2020). Monitoring the expression of both high (*BnaNRT2.1*) and low-affinity NO_3_^–^ transporters (*BnaNPF7.3*) showed suppression of their expression in the roots under K deprivation. We believe that the lack of K diminished the uptake of NO_3_^–^ since K is a counter ion for the uptake of NO_3_^–^ (Drechsler et al., 2015). This is supported by lower uptake of NO_3_^–^ in both roots and shoots and highlights the fact that under K starvation, the uptake of NO_3_^–^ is more impacted. Moreover, it was previously shown that N translocation to the shoots is also tightly regulated at the transcriptional level in response to K level. Indeed, *NRT1.5* transporter, which is involved in NO_3_^–^ xylem loading, is downregulated under low K and would contribute to the adjustment of K^+^/NO_3_^–^ levels depending on K availability (Lin et al., 2008; Li et al., 2017). Moreover, the kinase, CIPK23 is involved in the regulation of both K^+^ and NO_3_^–^ uptake, through the activation of AKT1, HAK5 transporters, and the modulation of NRT1.1 activity (Raddatz et al., 2020).

### Potassium Deficiency Modulated the Changes in Amino Acid and Substantially Increased Put Concentration in *B. napus*

Potassium is the most abundant inorganic cation in plant cells (Dreyer and Uozumi, 2011). There is no doubt about the vital role of K in plant metabolism, because K deficiency affects the contents of primary and secondary metabolites (Amtmann and Armengaud, 2009). Interestingly, our results showed coordination between different amino acids and the polyamine, Put which are known to be involved in abiotic stress responses in plants. In general, a decrease of negatively charged amino acids and an increase of positively charged amino acids have been observed in different crop species exposed to K deprivation in order to restore, at least partially, the electric charge balance (Armengaud et al., 2009; Zeng et al., 2018). Consistent with these results, we observed a decrease in glutamic acid in the shoots and an increase in histidine and lysine in the roots. Surprisingly, in the present study, the level of arginine, a positively charged amino acid, decreased nearly twofold in both the roots and shoots after 7 and 14 days of K deficiency. Another novel observation in this study was an increase in the negatively charged aspartic acid. Both arginine and aspartic acid are involved in the synthesis of other important metabolites. The decrease of arginine observed here could be related to the strong increase of Put in both the roots and the shoots. Put is synthesized from ornithine, either *via* the direct route involving ornithine decarboxylase or the indirect route that involves arginine decarboxylase depending on the plant species (Cui et al., 2020b). The indirect pathway has also been suggested to run in response to different abiotic stresses (Masgrau et al., 1997). In general, Put is accepted to be a good low-K biomarker and several studies have shown that an increase in its level upon K deficiency, and it has been suggested to participate in the regulation of H^+^-ATPases activity and vacuolar transporters to maintain Ca^2+/^K^+^ balance in one hand and to boost energy metabolism on the other hand (Ali et al., 2018; Cui et al., 2020b). Another explanation for the higher accumulation of Put in the present work under K deficiency could be due to an increase in the level of both Ser and Gly particularly in the shoots. Gly has been reported to play a pivotal role in increasing the stress tolerance in crop plants (Tripathi et al., 2013). It acts as a direct source of NH_4_^+^ and is also involved in increasing the level of amino acid, Ser (Schiller et al., 1998; Waditee et al., 2005) which might influence Put synthesis. Furthermore, the increase of aspartic acid observed in this study could also be related to the increased GS/GOGAT/GDH cycle. In a study on *Arabidopsis*, Armengaud et al. (2009) have shown an increase of GS/GOGAT/GDH activity in response to K deficiency which in turn adjusted N assimilation and also mediated the increase of N-rich amino acids. In agreement with this study, we observed an increase in certain GS-GOGAT-related amino acids, such as glutamine and asparagine, and a general increase in N-rich amino acids. It seems that rapeseed depends less on the decrease of negatively charged amino acid and uses the synthesis of Put to cope with K deprivation.

Additionally, here, we observed an increase in all the three aromatic amino acids after K withdrawal, especially tyrosine with a ninefold increase at 7 days and 13-fold at 14 days. Tyrosine, phenylalanine, and tryptophan serve as precursors for several major classes of secondary metabolites, including phenylpropanoids, flavonoids, and isoquinoline alkaloids. Secondary metabolites play an important role in the response and adaptation of plants to abiotic stress (Ramakrishna and Ravishankar, 2011; Kaur and Ganjewala, 2019). Diverse secondary metabolites could also be implicated in response to K deficiency. A study on barley also showed an increase of phenylalanine ammonia lyase protein, a key enzyme in the phenylpropanoid pathway, in low K condition (Zeng et al., 2018). Another study on oil palm leaves showed dopamine, which is derived from tyrosine, is among the most accumulated metabolites in low K condition (Cui et al., 2020a). Further analysis of specific classes of secondary metabolites by liquid chromatography (LC) and mass spectrometry (LC-MS) analysis will be interesting for a better understanding of rapeseed metabolic reprogramming and its response to K deficiency.

We also observed a general increase in the levels of soluble carbohydrates in both the roots and the shoots. The increase in the concentration of soluble carbohydrates was already shown in response to K in several plants, such as *Arabidopsis* (Armengaud et al., 2009), barley (Zeng et al., 2018), and tomato (Sung et al., 2015). The direct inhibition of pyruvate kinase which requires K as a cofactor could explain the accumulation of soluble carbohydrates due to the blockage of glycolysis (Amtmann and Armengaud, 2009). K deficiency could also impact the Suc concentration in the roots as the Suc loading into the phloem is stimulated by K (White and Karley, 2010; Gajdanowicz et al., 2011). This does not seem to be the case in rapeseed as we observed a similar or higher level of Suc in the roots compared to the shoots after K withdrawal. Altogether, these results indicate that rapeseed is very sensitive to K deprivation and synthesize Put at the expense of arginine and other amino acids, such as Gly and Ser to mitigate the lack of K.

### K Deprivation Elevates ABA Accumulation and Modulates the Expression of a Key Gene Involved in ABA Biosynthesis

Phytohormones have been considered to be the major signal in plant responses to K deficiency. Among the phytohormones, ethylene, as an important signal molecule in plant responses to K^+^ deficiency, stimulates reactive oxygen species (ROS) generation and root morphological changes (Jung et al., 2009). There is also evidence highlighting that cytokinins negatively regulate low K response (Nam et al., 2012). However, in the present study, the levels of these phytohormones were not consistent at least in our experimental condition. ABA is also considered as a plant stress hormone and plays an important role in response to abiotic stresses (Schraut et al., 2005). An induction of ABA under nutrient deficiencies is known (Schraut et al., 2005). In this context, the changes in ABA level have been shown under N (Zakari et al., 2020) and P deficiencies (Trull et al., 1997). In fact, there is a strong cross-talk between K nutrition and ABA, particularly at the level of guard cell in the stomata. ABA induces changes in specific ion channels at both the plasmalemma and the tonoplast, leading to efflux of both K and anions at both membranes (MacRobbie, 1998). An augmentation in the levels of ABA has also been reported in the flag leaves of wheat exposed to K deprivation, supporting the cross-talk between ABA and K deficiency signaling (Haeder and Beringer, 1981). Consistent with the earlier reported observations (Peuke et al., 2002; Schraut et al., 2005), we observed an increase in ABA levels in the roots in response to K starvation. However, unlike the previous reports which highlighted either a small increase or no changes in the level of ABA in the shoots (Peuke et al., 2002), the ABA level increased at least twofold in K-deficient plants compared to ample K condition. The possible scenario in which ABA levels in the shoot increased in K-deficient rapeseed plants could be due to a reduction in S uptake and translocation which was imposed by K deficiency. Indeed, ABA biosynthesis and S metabolism probably interplay during abiotic stress. As S-based amino acid, cysteine serves as a precursor (as a S donor) for the sulfuration of molybdenum cofactor, acting as a cofactor for the last reaction step of ABA biosynthesis (Malcheska et al., 2017). Moreover, it was shown in *Arabidopsis* that low sulfate increased the expression of *NCED3* which catalyzes the rate-limiting step in ABA biosynthesis pathway (Cao et al., 2014). To verify whether ABA levels might be affected due to the reduction of S and is associated with enhanced ABA biosynthesis, transcript levels of *BnaNCED3* were determined and found to strongly respond to K stress especially when K deficiency persisted for 2 weeks. Nevertheless, the exact mechanism by which K deficiency reduces the S level and its relationship with ABA metabolism need to be further investigated.

Another possible scenario in which the shoots ABA levels increased in response to K deficiency might be because of an increase in the level of Put. A positive feedback loop between ABA and polyamines has already been suggested (Pál et al., 2018). In a study on *Arabidopsis*, it was shown that ABA modulates polyamine metabolism at transcriptional and metabolite levels in response to water stress (Alcázar et al., 2006). In another work, using mutants of the genes involved in Put synthesis, it was shown that Put controls the levels of ABA in response to low temperature by modulating ABA biosynthesis and gene expression (Cuevas et al., 2008). Recently, a cross-talk between polyamine, ABA, and proline has also been reported in wheat seedlings exposed to osmotic stress (Pál et al., 2018).

It was thus concluded that the K deprivation in rapeseed induces a massive accumulation of the free polyamine, Put which consequently induces ABA biosynthesis due to higher induction of *BnaNCED3*.

## Conclusion

**FIGURE 9:**
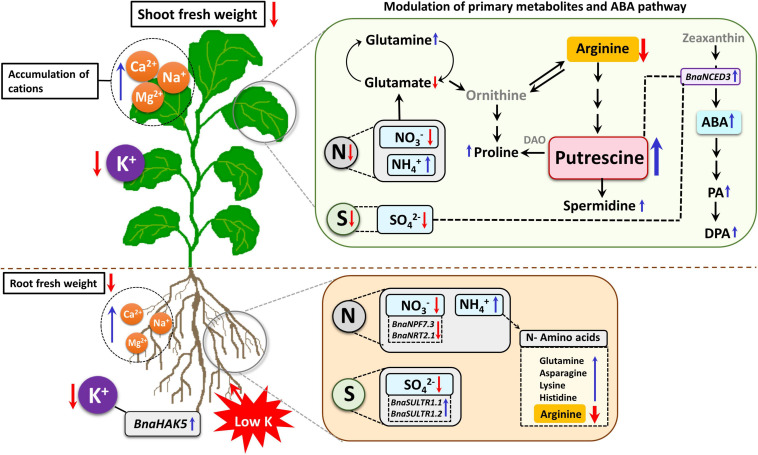
Schematic model illustrating the response of rapeseed plants to K deprivation. K deprivation systematically affects K concentration in both roots and shoots of rapeseed which consequently induces *BnaHAK5* transporter gene. Lack of K further modulates mineral nutrient status by increasing Ca, Mg, and Na levels in both roots and shoots. Moreover, concentration of nutrients, such as N and S also declined, and specific transcriptional changes were observed in their transporter genes. In both roots and shoots, major metabolic adjustments led to differential accumulation of amino acids in order to maintain the electric charge balance. These changes in amino acid levels eventually contributed to higher accumulation of polyamine putrescine (Put) which is known to interact with ABA through the induction of *BnaNCED3* gene and is also involved in proline accumulation through its catabolism *via* diamine oxidase (DAO). (Red arrows = decrease/downregulation, blue arrows = increase/upregulation, text in gray = not measured in this study, dashed arrows = hypothesized regulation).

In the present work, we demonstrated that the K deprivation changes nutritional balance, influences the levels of primary metabolites and phytohormone ABA, and modulates the key gene involved in ABA biosynthesis (Figure 9). Lower availability of K reduced the uptake and transport of K within the plant which was accompanied by a growth reduction in *B. napus* and induction in the expression level of the high-affinity K transporter, *BnaHAK5*. Moreover, the K stress reduced the concentrations of S and N and transcriptionally modulated the genes involved in their transport. Interestingly, K deficiency modulated primary metabolites in rapeseed plants which led to the substantial accumulation of Put to cope with severe K deficiency. This was supported by the lower accumulation of arginine as a positively charged amino acid and an increase in the levels of both Gly and Ser which all contributed to Put synthesis. Additionally, K-deficient rapeseed plants seem to cope with K stress by inducing *BnaNCED3* gene which was accompanied by higher levels of ABA. We believe that the K-starved rapeseed plants utilize polyamine accumulation and mainly Put accumulation as K-stress tolerance mechanism.

## Data Availability Statement

The original contributions presented in the study are included in the article/Supplementary Material, further inquiries can be directed to the corresponding author/s.

## Author Contributions

SH and J-CY conceived the experiment. SH supervised the experiment. SH and ER designed the experiment and wrote the manuscript. ER conducted the experiment and performed all the statistical analysis. ER and NA performed and supervised the gene expression analysis, respectively. LJ supervised the hormone and metabolite analysis. All authors contributed critically for revising the manuscript.

## Conflict of Interest

The authors declare that the research was conducted in the absence of any commercial or financial relationships that could be construed as a potential conflict of interest.

## Publisher’s Note

All claims expressed in this article are solely those of the authors and do not necessarily represent those of their affiliated organizations, or those of the publisher, the editors and the reviewers. Any product that may be evaluated in this article, or claim that may be made by its manufacturer, is not guaranteed or endorsed by the publisher.
