# Supplementary material for: K Deprivation Modulates the Primary Metabolites and Increases Putrescine Concentration in Brassica napus
Source: Front Plant Sci. 2021 Aug 13;12:681895. doi: 10.3389/fpls.2021.681895 (PMC8409508; doi:10.3389/fpls.2021.681895)
Supplement: Supplementary file 1 [file Presentation_1.pptx]

## Slide 1
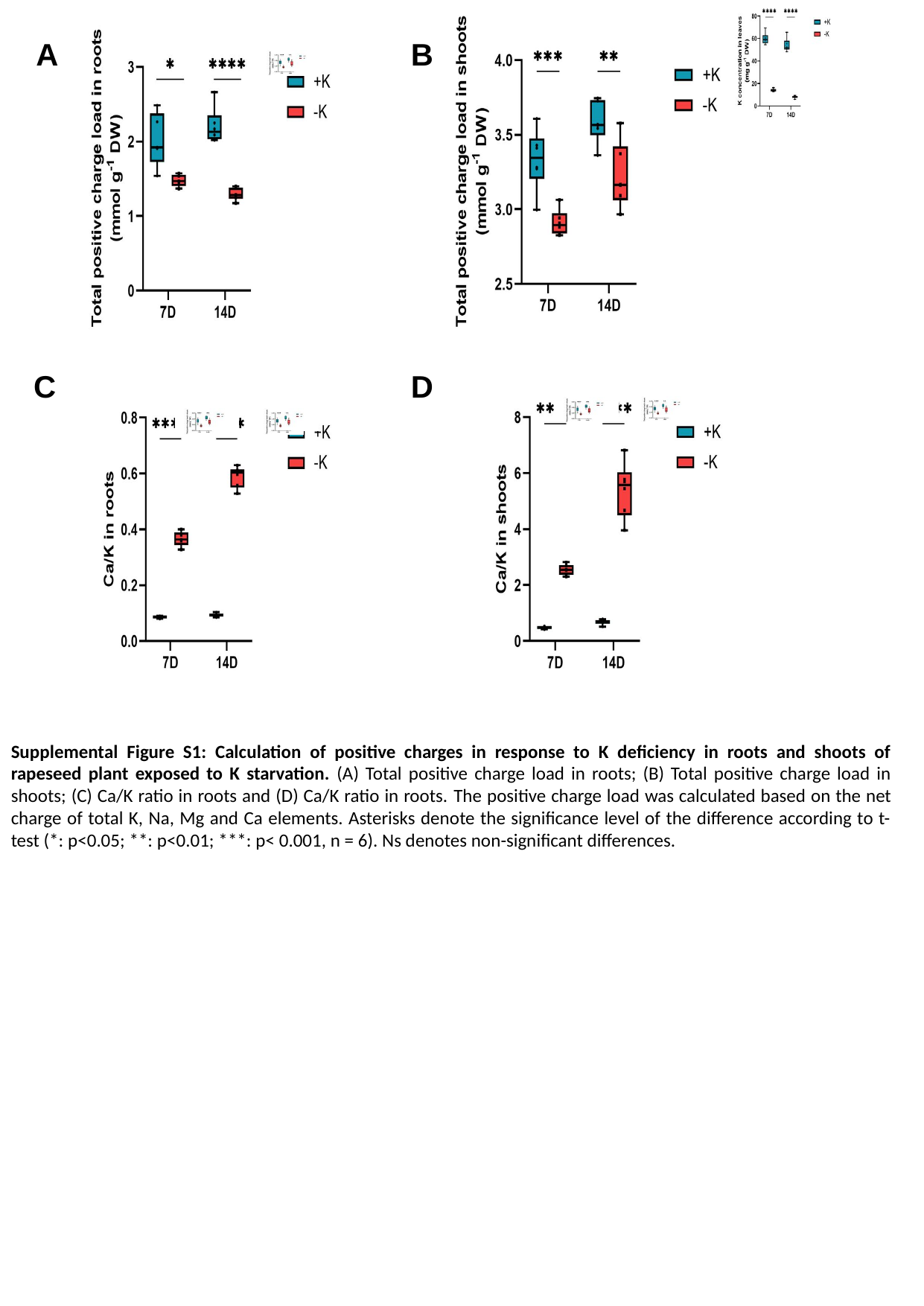

A
B
C
D
Supplemental Figure S1: Calculation of positive charges in response to K deficiency in roots and shoots of rapeseed plant exposed to K starvation. (A) Total positive charge load in roots; (B) Total positive charge load in shoots; (C) Ca/K ratio in roots and (D) Ca/K ratio in roots. The positive charge load was calculated based on the net charge of total K, Na, Mg and Ca elements. Asterisks denote the significance level of the difference according to t-test (*: p<0.05; **: p<0.01; ***: p< 0.001, n = 6). Ns denotes non-significant differences.
