# Supplementary material for: K Deprivation Modulates the Primary Metabolites and Increases Putrescine Concentration in Brassica napus
Source: Front Plant Sci. 2021 Aug 13;12:681895. doi: 10.3389/fpls.2021.681895 (PMC8409508; doi:10.3389/fpls.2021.681895)
Supplement: Supplementary file 2 [file Presentation_2.pptx]

## Slide 1
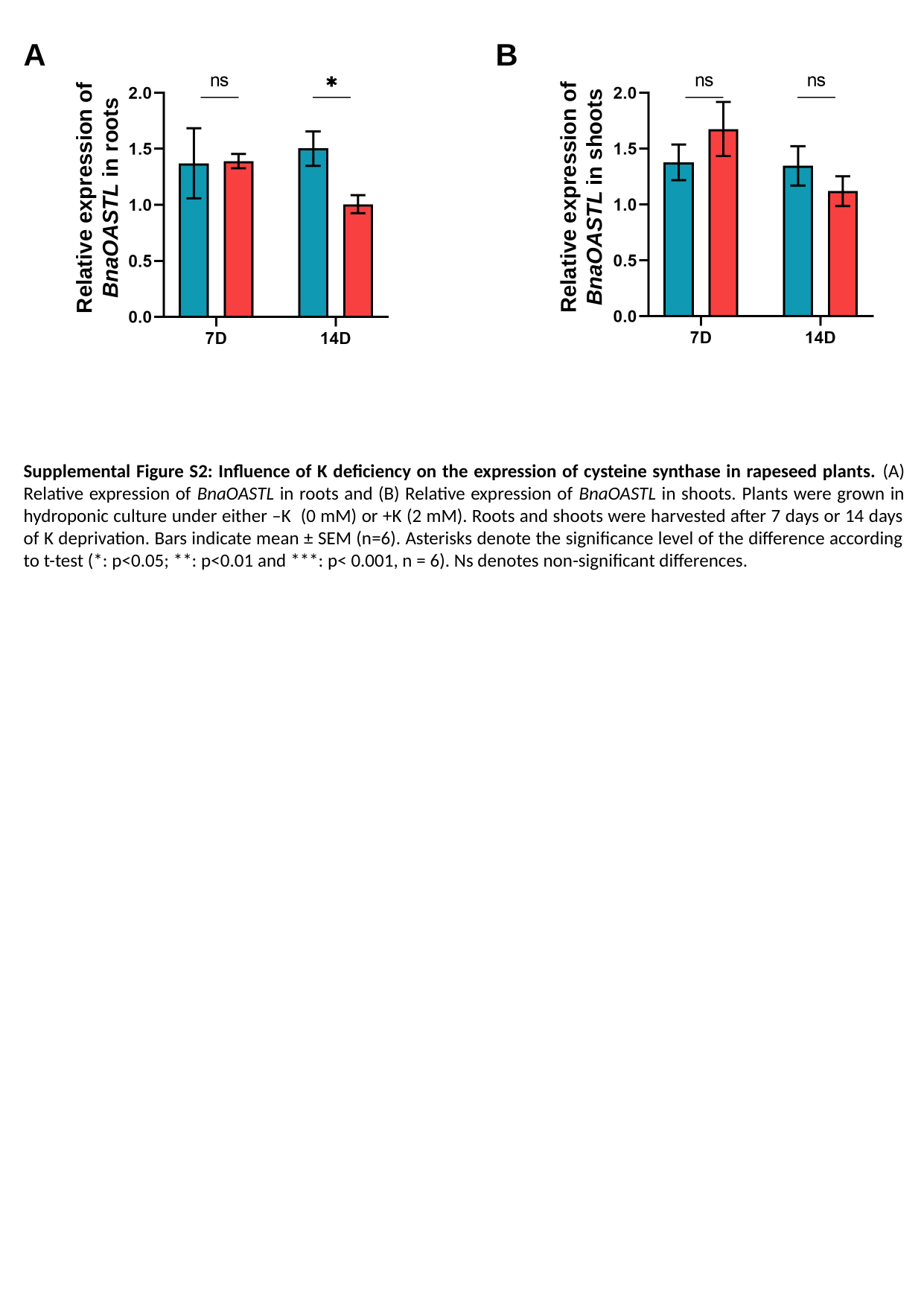

A
B
Relative expression of BnaOASTL in shoots
Relative expression of BnaOASTL in roots
Supplemental Figure S2: Influence of K deficiency on the expression of cysteine synthase in rapeseed plants. (A) Relative expression of BnaOASTL in roots and (B) Relative expression of BnaOASTL in shoots. Plants were grown in hydroponic culture under either –K (0 mM) or +K (2 mM). Roots and shoots were harvested after 7 days or 14 days of K deprivation. Bars indicate mean ± SEM (n=6). Asterisks denote the significance level of the difference according to t-test (*: p<0.05; **: p<0.01 and ***: p< 0.001, n = 6). Ns denotes non-significant differences.
