# Supplementary material for: K Deprivation Modulates the Primary Metabolites and Increases Putrescine Concentration in Brassica napus
Source: Front Plant Sci. 2021 Aug 13;12:681895. doi: 10.3389/fpls.2021.681895 (PMC8409508; doi:10.3389/fpls.2021.681895)
Supplement: Supplementary file 3 [file Table_1.docx]

**Supplemental Table S1:** List of the primers used for qRT-PCR.

| **Gene** | **Forward primer** | **Reverse primer** | **Amplicon size** |
| --- | --- | --- | --- |
| *BnaAKT1* | TACTGCGGACTTGGTTGTTC | ACCTATCTCCCCTATAATGTCTCC | 85 |
| *BnaHAK5* | TCACCCTTGTCGCACTTCT | GTTCTCTATCTTCTGGCTCTTGG | 147 |
| *BnaNPF7.3* | TGGGCATTGGACTTGTGATTG | TGAGCGAGTATTGTGGAACCT | 150 |
| *BnaNRT2.1* | GTATGTTCTTGCCTCCTTCCAC | GTTCTTCTGCTTCTCCTGCTC | 92 |
| *BnaSULTR1.1* | GGTCTCTTGATTTCCGTGGTG | TTCCGATACACATTCGTCCTTG | 107 |
| *BnaSULTR1.2* | TCAGCCATTTACTTCTCCAACTC | TGCTTTCACCTTCTCTTCTTCC | 84 |
| *BnaSULTR2.1* | GTCTGCCTTGTTGTGCTTTG | TCACCTTCTTCTTCCTGCCT | 81 |
| *BnaSULTR2.2* | ACCAATGCTGAAACACGAGG | CGACTTGACCGAGATGTGGA | 109 |
| *BnaOASTL* | CCCTGCCAACCCAAAGATAC | ACCAATCCCAGAAACAAATCCA | 94 |
| *BnaNCED3* | TCCTGCTCTCCATTTCCCC | TTGGTGTCGGATTCTTTGGTT | 81 |
| *BnaACT7* | TGAGAGATTCCGTTGCCCT | CACCACTGAGGACGATGTTTC | 149 |
| *BnaTIP41* | GGCACGATTCTCACTTCTCTC | CATTCTCGCCAAAGACCATTTC | 88 |
| *BnaPP2A* | TCCTTTTGTTGGAGGGGTTG | CTCTCTCACGCAGGTCTCTT | 98 |
| *BnaACT2* | ACAGTGTCTGGATCGGTGGTTC | TGCCTCATCATACTCAGCCTTG | 86 |
