# Supplementary material for: K Deprivation Modulates the Primary Metabolites and Increases Putrescine Concentration in Brassica napus
Source: Front Plant Sci. 2021 Aug 13;12:681895. doi: 10.3389/fpls.2021.681895 (PMC8409508; doi:10.3389/fpls.2021.681895)
Supplement: Supplementary file 4 [file Table_2.docx]

|  |  | **Roots** | | | |  | **Shoots** | | | |
| --- | --- | --- | --- | --- | --- | --- | --- | --- | --- | --- |
|  |  | **7 days** | | **14 days** | |  | **7 days** | | **14 days** | |
|  |  | **+K** | **-K** | **+K** | **-K** |  | **+K** | **-K** | **+K** | **-K** |
| **Amino acids** | **Alanine** | 0.29 ± 0.01 | 0.31 ± 0.02 | 0.45 ± 0.15 | 0.25 ± 0.05 |  | 0.68 ± 0.06 | 0.44 ± 0.05 | 0.90 ± 0.07 | 0.57 ± 0.04 |
|  | **Arginine** | 0.73 ± 0.17 | 0.48 ± 0.29 | 1.10 ± 0.09 | 0.60 ± 0.06 |  | 1.61 ± 0.17 | 0.77 ± 0.05 | 1.61 ± 0.15 | 0.68 ± 0.14 |
|  | **Asparagine** | 0.98 ± 0.17 | 2.88 ± 0.52 | 0.84 ± 0.09 | 5.54 ± 0.34 |  | 0.95 ± 0.06 | 1.31 ± 0.20 | 0.94 ± 0.13 | 2.19 ± 0.35 |
|  | **Aspartate** | ND | 0.21 ± 0.05 | ND | 0.19 ± 0.05 |  | ND | ND | 0.10 ± 0.03 | 0.58 ± 0.10 |
|  | **GABA** | 0.44 ± 0.03 | 0.46 ± 0.04 | 0.55 ± 0.09 | 0.53 ± 0.09 |  | 0.05 ± 0.01 | 0.06 ± 0.01 | 0.07 ± 0.03 | 0.06 ± 0.01 |
|  | **Glutamate** | 0.79 ± 0.14 | 0.81 ± 0.11 | 0.87 ± 0.06 | 0.83 ± 0.16 |  | 0.80 ± 0.04 | 0.55 ± 0.03 | 0.82 ± 0.08 | 0.70 ± 0.08 |
|  | **Glutamine** | 1.64 ± 0.34 | 2.93 ± 0.32 | 1.55 ± 0.30 | 1.81 ± 0.16 |  | 3.06 ± 0.29 | 3.23 ± 0.53 | 2.89 ± 0.30 | 7.51 ± 1.15 |
|  | **Glycine** | 0.16 ± 0.02 | 0.17 ± 0.03 | 0.14 ± 0.02 | 0.14 ± 0.02 |  | 0.24 ± 0.04 | 1.00 ± 0.34 | 0.41 ± 0.06 | 2.65 ± 1.02 |
|  | **Histidine** | 0.14 ± 0.03 | 0.31 ± 0.05 | 0.15 ± 0.02 | 0.28 ± 0.04 |  | ND | ND | ND | ND |
|  | **Isoleucine** | 0.11 ± 0.01 | 0.39 ± 0.09 | 0.12 ± 0.02 | 0.38 ± 0.09 |  | 0.08 ± 0.01 | 0.08 ± 0.01 | 0.08 ± 0.00 | 0.12 ± 0.02 |
|  | **Leucine** | 0.09 ± 0.01 | 0.35 ± 0.09 | 0.10 ± 0.02 | 0.36 ± 0.09 |  | ND | ND | ND | ND |
|  | **Lysine** | 0.13 ± 0.02 | 0.50 ± 0.13 | 0.15 ± 0.03 | 0.50 ± 0.14 |  | ND | ND | ND | ND |
|  | **Phenylalanine** | 0.09 ± 0.01 | 0.20 ± 0.06 | 0.10 ± 0.01 | 0.19 ± 0.05 |  | 0.10 ± 0.01 | 0.12 ± 0.01 | 0.10 ± 0.01 | 0.21 ± 0.03 |
|  | **Proline** | 0.99 ± 0.14 | 0.97 ± 0.04 | 1.09 ± 0.07 | 0.92 ± 0.13 |  | 0.30 ± 0.00 | 0.39 ± 0.04 | 0.31 ± 0.02 | 0.67 ± 0.14 |
|  | **Serine** | 0.61 ± 0.09 | 0.81 ± 0.12 | 0.62 ± 0.04 | 0.59 ± 0.08 |  | 1.58 ± 0.31 | 2.16 ± 0.09 | 1.77 ± 0.12 | 3.54 ± 0.59 |
|  | **Threonine** | 0.45 ± 0.09 | 0.46 ± 0.11 | 0.52 ± 0.05 | 0.50 ± 0.13 |  | 0.46 ± 0.03 | 0.28 ± 0.03 | 0.48 ± 0.05 | 0.39 ± 0.07 |
|  | **Tryptophan** | 0.11 ± 0.01 | 0.22 ± 0.03 | 0.13 ± 0.02 | 0.27 ± 0.04 |  | ND | ND | ND | ND |
|  | **Tyrosine** | 0.09 ± 0.03 | 0.83 ± 0.16 | 0.09 ± 0.01 | 1.16 ± 0.15 |  | ND | 0.98 ± 0.19 | ND | 2.68 ± 0.52 |
|  | **Valine** | 0.19 ± 0.01 | 0.70 ± 0.14 | 0.20 ± 0.02 | 0.66 ± 0.15 |  | 0.13 ± 0.01 | 0.15 ± 0.01 | 0.12 ± 0.01 | 0.23 ± 0.04 |
|  | **Methionine** | 3.24 ± 1.00 | 2.09 ± 0.46 | 5.16 ± 1.02 | 2.99 ± 0.47 |  | 8.43 ± 2.47 | 7.12 ± 1.48 | 3.92 ± 0.90 | 2.22 ± 0.58 |
| **Carbohydrates** | **Glucose** | 5.96 ± 1.36 | 17.09 ± 1.32 | 3.70 ± 0.87 | 8.18 ± 1.40 |  | 5.18 ± 1.55 | 20.40 ± 2.64 | 3.66 ± 1.81 | 15.91 ± 3.39 |
|  | **Fructose** | 1.54 ± 0.27 | 6.40 ± 0.66 | 1.53 ± 0.31 | 4.28 ± 1.08 |  | 2.88 ± 0.79 | 13.51 ± 1.69 | 3.11 ± 1.26 | 13.24 ± 2.66 |
|  | **Saccharose** | 11.68 ± 0.93 | 30.11 ± 4.71 | 14.70 ± 3.52 | 20.84 ± 3.05 |  | 13.10 ± 1.54 | 18.36 ± 2.12 | 10.42 ± 2.16 | 12.87 ± 2.29 |
| **Polyamines** | **Putrescine** | 5.93 ± 0.74 | 62.42 ± 7.42 | 4.86 ± 1.13 | 78.19 ± 13.44 |  | 0.86 ± 0.23 | 62.15 ± 13.74 | 1.35 ± 0.26 | 150.91 ± 26.52 |
|  | **Spermidine** | 15.27 ± 4.28 | 19.88 ± 1.58 | 12.17 ± 1.51 | 17.13 ± 0.58 |  | 23.49 ± 1.72 | 29.07 ± 3.24 | 21.78 ± 1.54 | 25.24 ± 2.22 |
|  | **Spermine** | 6.63 ± 2.55 | 2.36 ± 0.14 | 4.16 ± 0.94 | 0.46 ± 0.21 |  | 25.32 ± 2.46 | 15.16 ± 1.30 | 26.69 ± 1.39 | 12.28 ± 1.97 |

**Supplemental Table S2: Influence of K deficiency on the metabolites in rapeseed roots and shoots** Plants were grown in hydroponic culture under either K (0 mM) or +K (2 mM). Roots and shoots were harvested after 7 days or 14 days of K deprivation. Data are presented as mean ± SD. ND: non determined. All carbohydrates and amino acids are expressed in mg g^-1^ DW. Methionine and polyamines are expressed in µg g^-1^ FW.
